# Genetic characteristics and antimicrobial resistance of *Staphylococcus aureus* isolates from pig farms in Korea: emergence of *cfr*-positive CC398 lineage

**DOI:** 10.1186/s12917-024-04360-w

**Published:** 2024-11-01

**Authors:** Jun Bong Lee, Ji Hyun Lim, Ji Heon Park, Gi Yong Lee, Kun Taek Park, Soo-Jin Yang

**Affiliations:** 1 Department of Veterinary Microbiology, College of Veterinary Medicine and Research Institute for Veterinary Science, Seoul National University, Seoul, 08826 Korea; 2 Department of Molecular Microbiology and Immunology, Keck School of Medicine, University of Southern California, Los Angeles, CA USA; 3 Department of Biotechnology, Inje University, Gimhae, 50834 Korea

**Keywords:** *Staphylococcus aureus*, Pig farms, Clonal complex 398, Antimicrobial resistance, Linezolid resistance

## Abstract

**Background:**

Livestock-associated *Staphylococcus aureus* (LA-SA) has gained global attention because of its ability to colonize farm animals and transmit to the environment and humans, leading to symptomatic infections and the spread of antimicrobial resistance (AMR). In the last decade, numerous studies have reported a high prevalence of *S. aureus* clonal complex (CC) 398 in pig farms.

**Results:**

In this study, 163 *S. aureus* isolates were collected from healthy pigs (*n* = 110), farm environments (*n* = 42), and farm workers (*n* = 11), and their AMR profiles and epidemiological characteristics were analyzed. We identified 51 (31.3%) methicillin-resistant *S. aureus* (MRSA) and 112 (68.7%) methicillin-susceptible *S. aureus* (MSSA), with 161 (98.8%) isolates belonging to the CC398 lineage. The highest prevalence of *spa* type t571 was observed among the CC398 isolates. All 47 sequence type (ST) 398 MRSA isolates carried staphylococcal cassette chromosome *mec* (SCC*mec*) V, while four ST541 isolates carried SCC*mec* IV. High levels of resistance to commonly used antibiotics, including phenicols, quinolones, lincosamides, macrolides, aminoglycosides, and tetracyclines, have been observed on Korean pig farms. Notably, 21 *cfr*-positive CC398 isolates (four ST541-SCC*mec* IV MRSA and 17 ST398 MSSA) displaying increased resistance to linezolid were identified in healthy pigs.

**Conclusions:**

In summary, these findings suggest that the multidrug-resistant CC398 *S. aureus* lineage predominantly colonizes healthy pigs and farm environments in Korea. The emergence of *cfr*-positive *S. aureus* at human-animal interfaces presents a significant threat to food safety and public health.

**Supplementary Information:**

The online version contains supplementary material available at 10.1186/s12917-024-04360-w.

## Background

*Staphylococcus aureus* is an opportunistic pathogen responsible for a wide range of human diseases including skin infections, pneumonia, endocarditis, and osteomyelitis [1]. Methicillin-resistant *S. aureus* (MRSA), a leading cause of morbidity worldwide, has been restricted to the hospital- and community-associated infections [2]. However, a new subset of MRSA, known as livestock-associated MRSA (LA-MRSA) has also emerged worldwide over the past 20 years [3]. Recent studies have shown that farm animals are important reservoirs of both MRSA and methicillin-susceptible *S. aureus* (MSSA), and their transmission to veterinarians and farm workers is a growing concern [4, 5]. Recently, LA-MRSA isolates belonging to clonal complex (CC) 398 have gained significant attention due to their ability to easily cross host species boundaries [6, 7]. While pigs are the primary reservoirs of LA-MRSA CC398, colonization of other animals, including horses, cattle, poultry, and dogs, with the CC398 lineage has also been reported [8]. Since its first outbreak in the Netherlands [9], multidrug-resistant (MDR) LA-MRSA CC398 has been identified in pig farms and among farm workers worldwide, raising concerns regarding its zoonotic potential. LA-MRSA CC398 was characterized by (i) the presence of staphylococcal cassette chromosome *mec* (SCC*mec*) types V, (ii) the most prevalent staphylococcal protein A (*spa*) types t011, t018, and t034, and (iii) the presence of the tetracycline resistance gene, *tet*(M) [10].

Antibiotics such as β-lactams, phenicols, and tetracyclines are most frequently used in global pork production systems leading to a high prevalence of MDR LA-SA due to the selective pressure on pig microbiota [11]. Previous publications from our laboratory reported high levels of MDR phenotypes in MRSA (87.5%) and MSSA (58.5%) isolates collected from pig farms in Korea [12, 13]. Moreover, the occurrence of chloramphenicol-florfenicol resistance gene (*cfr*)-mediated linezolid resistance in sequence type (ST) 398 LA-MRSA isolated from pig farms was reported in Korea [14]. In addition to the β-lactam resistance associated with SCC*mec* elements, the *cfr*-mediated resistance to phenicols, lincosamides, oxazolidinones, pleuromutilins, and streptogramin A (PhLOPS_A_) is becoming a serious global health concern [15]. Therefore, it is crucial to investigate the clonal characteristics and antimicrobial resistance (AMR) profiles of LA-MRSA and LA-MSSA isolates from major livestock populations in Korea. In the present study, the major genetic characteristics and AMR profiles of both MRSA and MSSA isolates from pig farms were analyzed. Additionally, the prevalence of linezolid resistance and the genetic factors responsible for the resistance phenotype were examined in pig-associated *S. aureus* isolates.

## Methods

### Sample collection and *S. Aureus* isolation

*S. aureus* strains were isolated and identified as previously described [16]. Briefly, a total of 320 swab samples were obtained from 200 pigs (nasal swabs), 100 farm environmental sites (floor entry, floor exist, fence, drinking bottle, feed box, and ventilation fan), and 20 farm workers (inner side of gloves and boots) at 10 different pig farms located in five provinces of Korea including Gyeonggi (farms I and II), Chungcheong (farms III and IV), Gyeongsang (farms V and VI), Jeolla (farms VII and VIII), and Gangwon (farms IX and X) during 2023.

All collected swab samples were taken aseptically and inoculated into 3 mL of tryptic soy broth (BD Difco, Sparks, MD, USA) containing 10% sodium chloride and pre-enriched at 37℃ for 18–24 h. For *S. aureus* isolation, 10 µL of the enriched cultures were streaked onto Baird-Parker agar (BD Difco, Sparks, MD, USA) supplemented with potassium tellurite and egg yolk (BD Difco, Sparks, MD, USA) and incubated at 37℃ for 24–48 h. Plates were subsequently examined for putative *S. aureus* colonies displaying clear halos around the black colonies. Each putative isolate was identified using a matrix-assisted laser desorption ionization system (Bruker Daltonics, Bremen, Germany) and 16 S rRNA sequencing, as previously described [16]. Finally, confirmed *S. aureus* colonies were stored at -80℃ in 20% (v/v) glycerol stocks. For further experiments, frozen bacterial cell stocks were aseptically recovered on tryptic soy agar at 37℃ for 18 h, with single colonies were used in subsequent assays.

### Antimicrobial susceptibility test

Antimicrobial susceptibility assay for *S. aureus* isolates were performed using standard disc diffusion assays, as previously described [16]. The 12 antimicrobials agents tested included cefoxitin (FOX, 30 µg), chloramphenicol (CHL, 30 µg), ciprofloxacin (CIP, 5 µg), clindamycin (CLI, 2 µg), erythromycin (ERY, 15 µg), fusidic acid (FUS, 10 µg), gentamicin (GEN, 30 µg), mupirocin (MUP, 200 µg), rifampicin (RIF, 5 µg), trimethoprim-sulfamethoxazole (SXT, 23.73–1.25 µg), quinupristin-dalfopristin (SYN, 15 µg), and tetracycline (TET, 30 µg). *S. aureus* colonies grown on Müller-Hinton agar (BD Difco, Sparks, MD, USA) were suspended in 2 mL of distilled water to a McFarland standard of 0.5. Staphylococcal cell suspensions were spread onto MH agar plates using a sterilized swab, and antimicrobial discs (BD Difco, Sparks, MD, USA) were dispensed onto the plates within 15 min. The plated were incubated at 37℃ for 18 h, and susceptibility were interpreted according to guidelines of Clinical and Laboratory Standards Institute (CLSI, 2023). For linezolid susceptibility, minimum inhibitory concentration (MIC) was determined using a standard Etest (bioMérieux, Craponne, France). *S. aureus* ATCC 29,213 and ATCC 25,923 were used as reference strains for all antimicrobial susceptibility tests.

To analyze changes in the AMR pattern of *S. aureus* isolates from 2018 to 2023 in Korea, we compared our results with those of previous studies on 37 MRSA and 37 MSSA isolates from pig farms in 2018 [12, 13]. Pearson’s chi-square test was used to assess differences in AMR between groups of *S. aureus* isolates. Statistical analysis was performed using GraphPad Prism 5 (GraphPad Software Inc., Boston, MA, USA), and *P* < 0.05 was considered statistically significant. Annual sales data for six classes of antimicrobial agents (phenicols, quinolones, lincosamides, macrolides, aminoglycosides, and tetracyclines) in pig farms from 2018 to 2022 were sourced from Animal and Plant Quarantine Agency (APQA) reports in Korea [17].

### MLST and *spa* typing of *S. Aureus* isolates

Multilocus sequence typing (MLST) was performed as described previously [18]. Briefly, internal fragments of seven house-keeping genes (*arcC*, *aroE*, *glpF*, *gmk*, *pta*, *tpi*, and *yqiL*) were amplified, and the PCR products were analyzed via DNA sequencing (BIONICS, Seoul, Korea). Next, sequences were submitted to the SA MLST database (https://pubmlst.org/saureus/) to assign specific sequence types (ST).

To determine the *spa* type of *S. aureus* isolate, the short sequence repeats in the polymorphic X regions of the *spa* gene were analyzed as previously described [19]. Corresponding *spa* types were determined based on the variable number of tandem repeats using the SpaServer database (http://spa.ridom.de/).

### SCC*mec* typing and detection of *cfr* in *S. Aureus* isolates

Following the antimicrobial susceptibility test, all *S. aureus* isolates displaying resistance to FOX were subjected to *mecA* detection as previously described [20]. According to CLSI guidelines, FOX-resistant *S. aureus* isolates carrying *mecA* gene were defined as MRSA. SCC*mec* typing of MRSA isolates was performed using multiplex PCR to amplify the chromosomal cassette recombinase (*ccr*) and *mec* gene complexes (*mec*) as previously described [21]. SCCmec types were identified by comparing *ccr* and *mec* gene complexes with representative MRSA strains: *S. aureus* COL (SCC*mec* I), N315 (SCC*mec* II), 85/2082 (SCC*mec* III), MW2 (SCC*mec* IV), and *S. aureus* WIS (SCC*mec* V).

All MRSA and MSSA isolates were subjected to PCR for detection of *cfr* gene. Primer sequences used for *cfr* detection and sequencing are listed in Table S1. To identify genetic alterations within the promoter and open reading frame (ORF) sequences of *cfr* genes, sequencing primers were designed based on the *cfr*-harboring plasmid pSA12 genome sequences (GenBank accession no. CP049977) [22]. Full-length coding sequence and promoter sequence (523-nt upstream region of the start codon) of *cfr* were amplified from genomic DNA of *cfr*-positive *S. aureus* isolates for Sanger sequencing analysis (Bionics, Seoul, Korea).

## Results

### Prevalence of MRSA and MSSA in pig farms

A total of 163 *S. aureus* strains were isolated from 320 swab samples (50.9%) obtained from ten different pig farms in Korea (Table 1). *S. aureus* isolation rates were 55% (110/200) in pigs, 42% (42/100) in farm environments, and 55% (11/20) in farm workers. *S. aureus* isolation rates varied across provinces: Gyeonggi (25.0%), Chungcheong (28.1%), Gyeongsang (82.8%), Jeolla (79.7%), and Gangwon (39.1%). Out of 163 isolates, 51 (38 from pigs, ten from farm environments, and three from farm workers) were identified as MRSA (31.3%) based on the *mecA* gene presence and FOX resistance phenotype. The 112 MSSA strains were isolated from pigs (*n* = 72), farm environments (*n* = 32), and farm workers (*n* = 8).

**Table 1:** Prevalence of MRSA and MSSA in pig farms located in five provinces of Korea

Sample origin	Provinces					Total
	Gyeonggi	Chungcheong	Gyeongsang	Jeolla	Gangwon	
**MRSA (** ***n*** ** = 51)**						
Pigs	0/40	9/40 (22.5)	11/40 (27.5)	16/40 (40.0)	2/40 (5.0)	38/200 (19.0)
Farm environments	0/20	1/20 (5.0)	5/20 (25.0)	3/20 (15.0)	1/20 (5.0)	10/100 (10.0)
Farm workers	0/4	0/4	0/4	3/4 (75.0)	0/4	3/20 (15.0)
Total	0/64	10/64 (15.6)	16/64 (25.0)	22/64 (34.4)	3/64 (4.7)	51/320 (15.9)
**MSSA (** ***n*** ** = 112)**						
Pigs	9/40 (22.5)	6/40 (15.0)	26/40 (65.0)	20/40 (50.0)	11/40 (27.5)	72/200 (36.0)
Farm environments	5/20 (25.0)	1/20 (5.0)	9/20 (45.0)	8/20 (40.0)	9/20 (45.0)	32/100 (32.0)
Farm workers	2/4 (50.0)	1/4 (25.0)	2/4 (50.0)	1/4 (25.0)	2/4 (50.0)	8/20 (40.0)
Total	16/64 (25.0)	8/64 (12.5)	37/64 (57.8)	29/64 (45.3)	22/64 (34.4)	112/320 (35.0)

Data are shown as number of isolates/number of samples (%)

### Genetic characteristics of MRSA and MSSA isolates

**Fig. 1:**
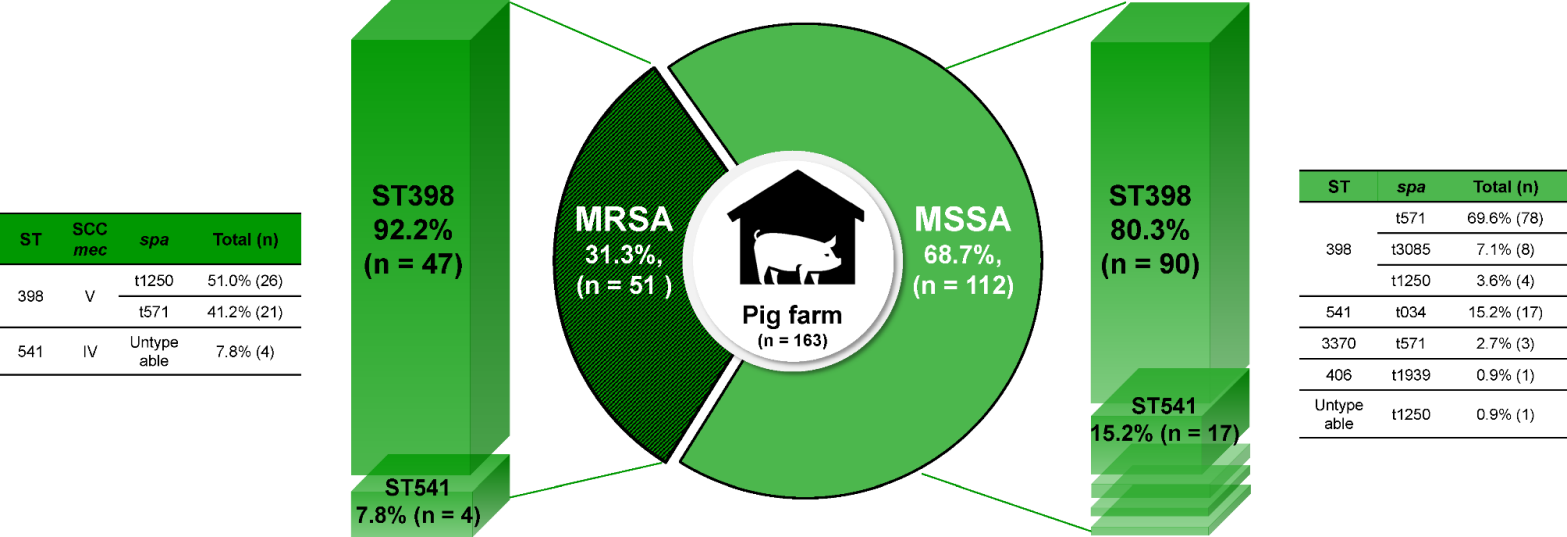
Genetic characteristics of MRSA and MSSA isolates. The genotypes of 51 MRSA and 112 MSSA isolates from pig farms were classified based on the allelic profiles of sequence type (ST) and staphylococcal protein A (*spa*). The staphylococcal cassette chromosome *mec* (SCC*mec*) type of MRSA isolates was determined using the polymerase chain reaction with primers specific to *ccr* and *mec* gene complexes encoded in the SCC*mec* element

All the 51 MRSA isolates belonged to the CC398 lineage: ST398 (47 isolates) and ST541 (*n* = 4) (Fig. 1). Similarly, 110/112 MSSA isolates belonged to ST398 (*n* = 90), ST541 (*n* = 17), and ST3370 (*n* = 3) within the CC398 lineage. Only two non-CC398 MSSA isolates (one ST406 and one non-typeable) were detected in Gyeonsang and Gangwon, respectively (Fig. S1). These results suggest that CC398 lineage predominate among pig-associated MRSA and MSSA isolates.

SCC*mec* typing analysis revealed that all 47 ST398 MRSA isolates carried SCC*mec* V, whereas all four ST541 MRSA isolates carried SCC*mec* IV for methicillin resistance (Fig. 1). The 47 ST398 MRSA-SCC*mec* V isolates were divided into two closely related *spa* types, t1250 (*n* = 26) and t571 (*n* = 21). Interestingly, all four ST541 MRSA-SCC*mec* IV isolates carried the non-typeable *spa* gene. Among the CC398 MSSA isolates, t571 *spa* type was most frequently associated with ST398 (78/90 isolates) and ST3370 (all three isolates). In contrast to the non-typeable *spa* types of ST541 MRSA isolates, all 17 ST541 MSSA isolates were *spa* type t034 (Fig. 1).

### Antimicrobial resistance of MRSA and MSSA isolates

**Fig. 2:**
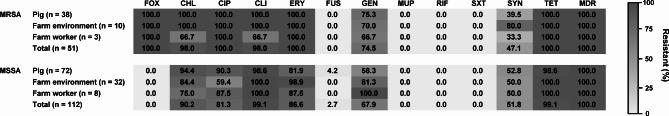
Heatmap showing the percentage of pig farm-originating MRSA and MSSA isolates resistant to antibiotics. Dark grey boxes represent the antibiotics which strains exhibited high resistance rates, whereas boxes in light grey represent low resistance rates. Bacteria resistant to three or more antibiotic classes were referred to as multidrug-resistant (MDR). FOX, cefoxitin; CHL, chloramphenicol; CIP, ciprofloxacin; CLI, clindamycin; ERY, erythromycin; FUS, fusidic acid; GEN, gentamicin; MUP, mupirocin; RIF, rifampicin; SXT, trimethoprim-sulfamethoxazole; SYN, quinupristin-dalfopristin; TET, tetracycline

Antimicrobial susceptibility analysis revealed 100% MDR frequency for both MRSA and MSSA isolates (Fig. 2). MRSA exhibited the highest resistance to CIP, ERY, and TET (100%), followed by CHL and CLI (98.0%), GEN (74.5%), and SYN (47.1%). MSSA isolates exhibited the highest resistance rates (99.1%) for CLI and TET, followed by CHL (90.2%), ERY (86.6%), CIP (81.3%), GEN (67.9%), and SYN (51.8%).

**Fig. 3:**
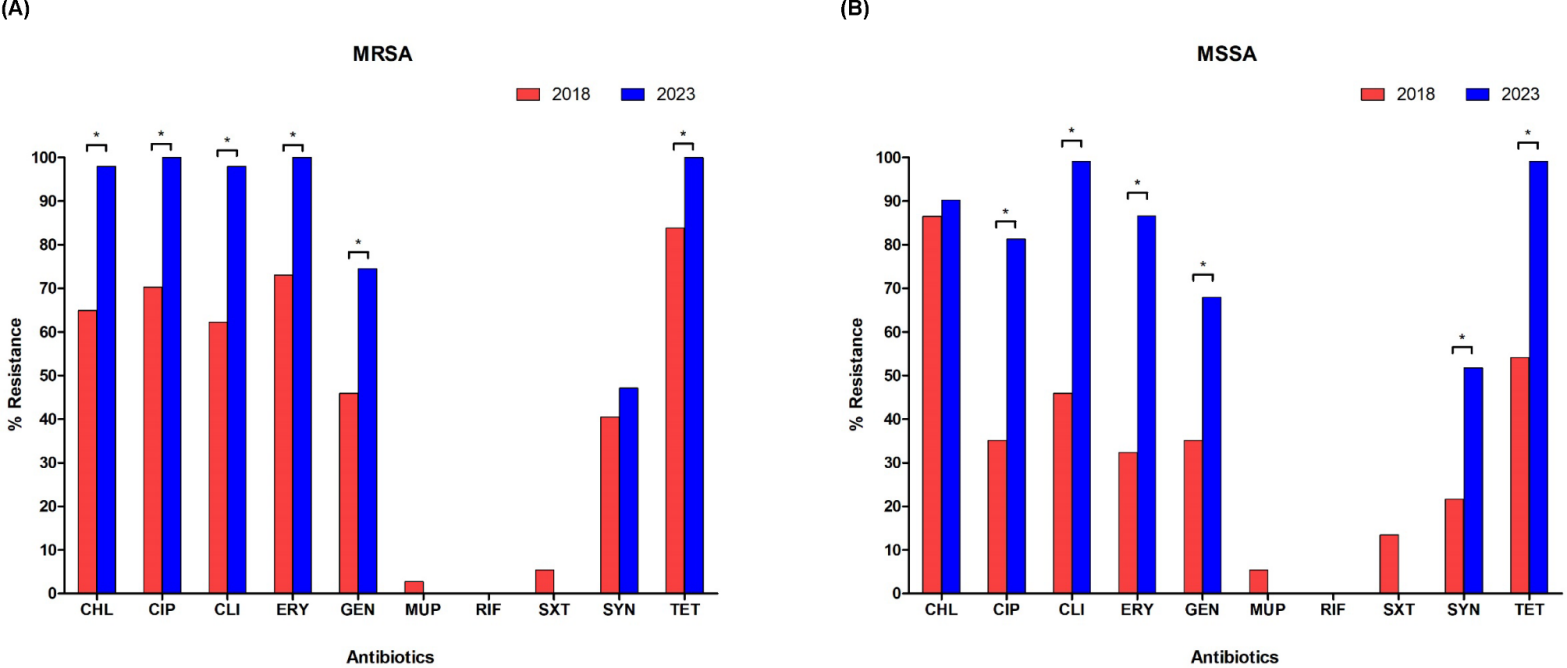
Altered antimicrobial resistance of MRSA and MSSA isolates from pig farms between 2018 and 2023. The percentages of resistance to 10 antibiotics in MRSA (**A**) and MSSA (**B**) isolates from pig farms in 2023 (blue bars) were compared with those in 2018 (red bars). CHL, chloramphenicol; CIP, ciprofloxacin; CLI, clindamycin; ERY, erythromycin; FUS, fusidic acid; GEN, gentamicin; MUP, mupirocin; RIF, rifampicin; SXT, trimethoprim-sulfamethoxazole; SYN, quinupristin-dalfopristin; TET, tetracycline. Statistical significance was obtained by Pearson’s chi-square test (^*^P < 0.05)

The AMR profiles of MRSA and MSSA isolates from in this study were compared with those of 74 the previously reported *S. aureus* (37 MRSA and 37 MSSA) from pig farms in 2018 [12, 13]. As shown in Fig. 3A, significantly higher levels of resistance to CHL (64.9% vs. 98.0%), CIP (70% vs. 100%), CLI (62.2% vs. 98.0%), ERY (73.0% vs. 100%), GEN (45.9% vs. 74.5%), and TET (83.8% vs. 100%) were observed in the MRSA isolates collected in 2023 (*P* < 0.05). Similarly, MSSA isolates collected in 2023 showed significantly higher resistance levels: CIP (35.1% vs. 81.3%), CLI (45.9% vs. 99.1%), ERY (32.4% vs. 86.6%), GEN (35.1% vs. 67.9%), SYN (21.6% vs. 51.8%), and TET (54.1% vs. 99.1%) (Fig. 3B) (*P* < 0.05).

### Identification of *cfr*-positive MRSA and MSSA isolates

**Fig. 4:**
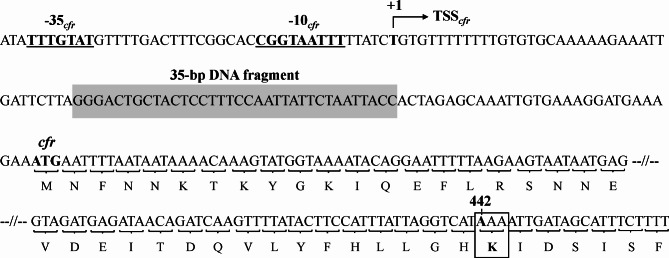
Genetic alterations associated with linezolid-susceptible phenotype in *cfr*-positive *S. aureus*. The black box depicts a 35-bp DNA fragment inserted into the 30-bp upstream of the start codon. Bent arrow indicates predicted transcription start site (TSS) for *cfr* gene. Putative − 35 and − 10 sequences for the potential TSS are underlined. TSS and promoter sequences for *cfr* were predicted using BPROM (Solovyev and Salamov, 2011) and BDGP (http://www.fruitfly.org/seq_tools/promoter.html) analysis tools

Of the 163 *S. aureus* isolates, 21 (12.9%) (four MRSA and 17 MSSA isolates) carried the *cfr* gene (Table 2). Notably, all four *cfr*-positive MRSA isolates were ST541 MRSA-SCC*mec* IV, while all 17 *cfr*-positive MSSA isolates were ST398 with *spa* t571. Despite carrying the *cfr* gene, these 21 *cfr*-positive MRSA and MSSA isolates exhibited susceptible phenotype to linezolid (linezolid MICs < 8 µg/ml). Sequencing analysis revealed that a point mutation (C to A) at position 442 in the *cfr* ORFs in the four ST541 MRSA isolates (Fig. 4). This change resulted in a glutamine-to-lysine substitution at amino acid 148 (Q148K). Additionally, as shown in Fig. 4, analysis of the *cfr* promoter sequence in the 17 *cfr*-positive ST398-t571 MSSA isolates revealed a 35-bp insertion sequence 30-bp upstream of the ATG start codon in the all 17 isolates (GenBank accession number PQ464589).

**Table 2:** Genotypes and AMR profiles of *cfr*-positive MRSA and MSSA isolates from pig farms

*S. aureus* isolates (No. of isolates)							
Genotype	Region	Origin	AMR profile	LZD MIC (µg/ml)			Genetic mutation of *cfr*
				1	2	4	
ST541-SCC*mec* IV (4)	Jeolla, farm VII	Pig (1)	FOX-CHL-CIP-CLI-ERY-SYN-TET (2)			(2)	Q184K mutation in *cfr* ORF
		Farm environment (2)	FOX-CHL-CIP-CLI-ERY-TET (1)			(1)	
		Farm worker (1)	FOX-CIP-ERY-SYN-TET (1)			(1)	
ST398-t571 (17)	Gyeong-sang, farm V	Pig (9)	CHL-CIP-CLI-ERY-GEN-SYN-TET (10)	(1)	(1)	(8)	35-bp insertion into *cfr* promoter region
		Farm environment (7)	CHL-CLI-ERY-GEN-SYN-TET (4)		(2)	(2)	
		Farm worker (1)	CHL-CIP-CLI-ERY-SYN-TET (1)			(1)	
			CHL-CLI-ERY-GEN-TET (1)			(1)	
			CHL-CIP-CLI-ERY-TET (1)	(1)			

LZD, linezolid; FOX, cefoxitin; CHL, chloramphenicol; CIP, ciprofloxacin; CLI, clindamycin; ERY, erythromycin; GEN, gentamicin; SYN, quinupristin-dalfopristin; TET, tetracycline

## Discussion

CC398 LA-MRSA is the predominant clonal lineage in Europe and North America, while CC9 is the dominant LA-MRSA in China [23, 24]. In addition to its high prevalence of the CC398 genotype among LA-MRSA isolates, the CC398 lineage becoming more prevalent among LA-MSSA isolates from Western and Central Europe [25, 26]. For example, Mroczkowska et al.. reported that 88.9% of MRSA and 62.7% of MSSA isolates from pigs, farm environments, and farm workers belonged to the CC398 genotype in Poland [27]. Previous studies from our laboratory revealed that 81.1% of MRSA and 32.0% of MSSA isolates obtained from Korean pig farms in 2018 belonged to the CC398 lineage [12, 13]. In the current study, all 51 MRSA isolates and 110/112 (98.2%) MSSA isolates were identified as CC398 (Fig. 1), indicating a rise in the prevalence of CC398 MRSA and CC398 MSSA on Korean pig farms over the last five years. CC398 LA-MRSA, particularly the ST398 clonal lineage, has been reported to colonize and infect humans especially those in close contact with farm animals [28, 29]. In this study, 15% (3/20) of farm workers were culture-positive for CC398 MRSA, which is lower than the prevalence rates among pig farm workers in Italy (21.6%) [30], Spain (57.9%) [31], the USA (45–49%) [32], and Canada (20%) [33]. Nonetheless, our results suggest that pigs are a substantial reservoir of both CC398 MRSA and CC398 MSSA for transmission to humans in Korea.

SCC*mec* V has most frequently been associated with CC398 LA-MRSA in many countries, including Korea [12, 16, 34]. In Australia, the CC398 MRSA-SCC*mec* V clone is now widespread in pig populations and causes staphylococcal sepsis in humans [7]. Similarly, all 47 ST398 MRSA isolates in this study harbored SCC*mec* V. Unexpectedly, four ST541 MRSA isolates carried SCC*mec* IV (Fig. 1), which has frequently been associated with ST72 community-associated MRSA in Korea [35]. ST398 MRSA-SCC*mec* IV isolates have previously been reported in humans [36] and farm animals [37, 38]. However, to our knowledge, this is the first report describing the occurrence of ST541 LA-MRSA harboring SCC*mec* IV in Korean pig farms. In contrast to the predominance of SCC*mec* V among CC398 LA-MRSA isolates, diverse *spa* types in CC398-type LA-MRSA and LA-MSSA isolates have been previously reported [10]. In this study, most CC398 MRSA isolates (92.2%) had the classical *spa* type t571 or a closely related type t1250 (Fig. 1) [10]. In agreement with previous studies [16], 82/90 (91.1%) ST398 MSSA isolates were typed as either t571 or t1250. However, unlike the untypeable *spa* types of the ST541 MRSA isolates, all 17 ST541 MSSA isolates were *spa* type t034. Additionally, 8/90 (8.9%) ST398 MSSA isolates had *spa* type t3085, which was not identified among the ST398 MRSA isolates. These results suggest that novel subpopulations of pig-associated MRSA and MSSA within the CC398 lineage occurred likely due to the acquisition of SCC*mec* IV and increased diversity of *spa* types.

From 2018 to 2023, phenicols, marcolides, and tetracyclines were the most frequently used antibiotic classes on pig farms in Korea [17]. The frequent and prolonged use of these antibiotics in Korean pig farms have led to an increased selection of AMR isolates, such as MDR MRSA and MSSA. In the current study, 98% of MRSA isolates displayed CHL resistance phenotype, which was significantly higher than that reported in previous studies from Korea (64.9%) [12], Japan (43.2%) [39], Belgium (10.9%) [40], and Spain (35.6%) [41]. In line with this notion, increased consumption of florfenicol in pig farms of Korea may have promoted the resistance to CHL [42]. Based on veterinary antibiotic agent sales data in Korea [17], the use of antibiotic agents of veterinary importance, including macrolides (tylosin and tilmicosin), aminoglycosides (streptomycin and neomycin), tetracyclines (chlortetracycline), quinolones (enrofloxacin), and lincosamides (lincomysin) may also have caused the higher levels of AMR phenotypes in CC398 isolates collected in the current study compared to those of the isolates collected in 2018 (Fig. 3).

Since the first detection in a bovine *S*. *sciuri* isolate in 2000 [43], a new transferable *cfr* gene, which mediates resistance to PhLOPS_A_ antibiotic classes, has been identified in numerous livestock-associated staphylococci worldwide, including Korea [14, 44, 45]. Oxazolidinones such as linezolid and tedizolid are critical for treating human infections caused by MDR pathogens, and their use for veterinary purposes is strictly prohibited in most countries. However, because of the MDR phenotypes mediated by the *cfr* gene product, any one or more of PhLOPS_A_ antibiotics can exert selection pressure on *cfr*-positive staphylococci, even in the absence of oxazolidinones in livestock farms [46–48]. As shown in Table 2, 21/163 (12.9%) *cfr*-positive *S. aureus* isolates were detected, which is higher than the rates reported in previous studies in Korea (2.3-7.4%) [12, 13, 16], China (15.6%) [49], the Netherlands (0.5%) [50], and Portugal (1.8%) [51]. All four *cfr*-positive MRSA isolates were genotype ST541-SCC*mec* IV from Jeolla province, and all 17 *cfr*-positive MSSA were ST398-t571 from Gyeongsang province, indicating that the *cfr* gene was preferentially distributed in these genotypes of isolates. Although the majority of previous studies examining *cfr*-positive staphylococci have focused on MRSA isolates [22, 44, 50, 51], the higher prevalence of *cfr* gene in MSSA isolates (17/112, 15.2%) than in the MRSA isolates (4/51, 7.8%) suggests that nationwide monitoring of *cfr* is necessary for both MRSA and MSSA on livestock farms. When all the 21 *cfr*-positive isolates were PCR-screened for an additional linezolid resistance-associated gene, *optrA* [52], none of the isolates were positive for the *optrA*. However, all the 21 *cfr*-positive isolates carried *fexA* conferring resistance to phenicols [53], suggesting that *fexA* and *cfr* genes might be colocalized on a transferable plasmid [22, 54]. Interestingly, while the *cfr*-positive isolates displayed higher level of linezolid MICs compared to *cfr*-negative isolates (Table S2), all the 17 *cfr*-positive *S. aureus* isolates failed to show linezolid-resistance phenotype (linezolid MICs of ≥ 8 µg/ml). In line with a previous study from our laboratory [22], four *cfr*-positive MRSA isolates carried a Q148K mutation in *cfr* ORF (Table 2). However, none of the 17 *cfr*-positive MSSA isolates harbored the Q148K mutation. Instead, sequence analysis of the *cfr* promoter region revealed that all 17 *cfr*-positive MSSA isolates had a 35-bp insertion sequences 30 bp upstream of the ATG start codon (Fig. 4). It is speculated that the presence of 35-bp insertion sequences in the promoter region results in reduced expression of *cfr*, thus causing a linezolid-susceptible phenotype in the 17 *cfr*-positive MSSA isolates. This study is the first to report a 35-bp insertion sequences in *cfr* promoter region associated with a linezolid-susceptible phenotype, even in the presence of the wild-type *cfr* in *S. aureus* isolates. Collectively, these results suggest that there are two distinctive molecular pathways for linezolid-susceptibility in *cfr*-positive ST541-SCC*mec* IV MRSA and ST398-t571 MSSA isolates from Korean pig farms.

## Conclusion

In conclusion, our data suggest that: (i) CC398 lineages of LA-MRSA and LA-MSSA are predominantly colonize pig farms in Korea; (ii) the significantly higher prevalence of MDR CC398 strains contributed to the increased levels of AMR phenotypes, particularly the MDR phenotype, over the past 5 years in Korean pig farms; (iii) there are two distinct subpopulations of *cfr*-positive CC398 lineage, ST541-SCC*mec* IV and ST398-t571, that exhibit linezolid-susceptible phenotype; and (iv) two distinctive genetic factors, the Q148K mutation in *cfr* ORF and 35-bp insertion sequence in *cfr* promoter region, are responsible for the linezolid-susceptible phenotype in *cfr*-positive staphylococcal isolates.

## Electronic supplementary material

Below is the link to the electronic supplementary material.

Supplementary Material 1

Supplementary Material 2

Supplementary Material 3

## Data Availability

Data is provided within the manuscript or supplementary information files.

## Abbreviations

LA-SA: Livestock-associated Staphylococcus aureus
MRSA: Methicillin-resistant S. aureus
MSSA: Methicillin-susceptible S. aureus
AMR: Antimicrobial resistance
MDR: Multidrug-resistant
MLST: Multilocus sequence typing
ST: Sequence type
CC: Clonal complex
SCCmec: Staphylococcal cassette chromosome mec
ccr: Chromosomal cassette recombinase
spa: Staphylococcal protein A
cfr: Chloramphenicol-florfenicol resistance gene
PhLOPS_A_: Phenicols, lincosamides, oxazolidinones, pleuromutilins, and streptogramin A
MIC: Minimum inhibitory concentration
ORF: Open reading frame
FOX: Cefoxitin
CHL: Chloramphenicol
CIP: Ciprofloxacin
CLI: Clindamycin
ERY: Erythromycin
FUS: Fusidic acid
GEN: Gentamicin
MUP: Mupirocin
RIF: Rifampicin
SXT: Trimethoprim-sulfamethoxazole
SYN: Quinupristin-dalfopristin
TET: Tetracycline
CLSI: Clinical and Laboratory Standards Institute
