# Supplementary material for: Genetic characteristics and antimicrobial resistance of Staphylococcus aureus isolates from pig farms in Korea: emergence of cfr-positive CC398 lineage
Source: BMC Vet Res. 2024 Nov 1;20:503. doi: 10.1186/s12917-024-04360-w (PMC11529005; doi:10.1186/s12917-024-04360-w)
Supplement: Supplementary file 1 — Supplementary Material 1 [file 12917_2024_4360_MOESM1_ESM.docx]

**Table S2. MICs of linezolid against *cfr*-positive and -negative *S. aureus***

| Antibiotic | Isolate | Distribution (%) of MIC (µg/ml) | | | | | |
| --- | --- | --- | --- | --- | --- | --- | --- |
|  |  | 0.5 | 1 | 2 | 4 | 8 | 16 |
| Linezolid | *cfr* (+) |  | 9.5 | 14.3 | 76.2 |  |  |
|  | *cfr* (-) |  | 19.7 | 78.2 | 2.1 |  |  |

White fields represent range of dilutions tested for linezolid. MICs equal to or lower than the lowest concentration tested are underlined. The epidemiological cut-off value for linezolid is presented as a vertical line.
