# Supplementary figures and images for: Genetic characteristics and antimicrobial resistance of Staphylococcus aureus isolates from pig farms in Korea: emergence of cfr-positive CC398 lineage
Source: BMC Vet Res. 2024 Nov 1;20:503. doi: 10.1186/s12917-024-04360-w (PMC11529005; doi:10.1186/s12917-024-04360-w)

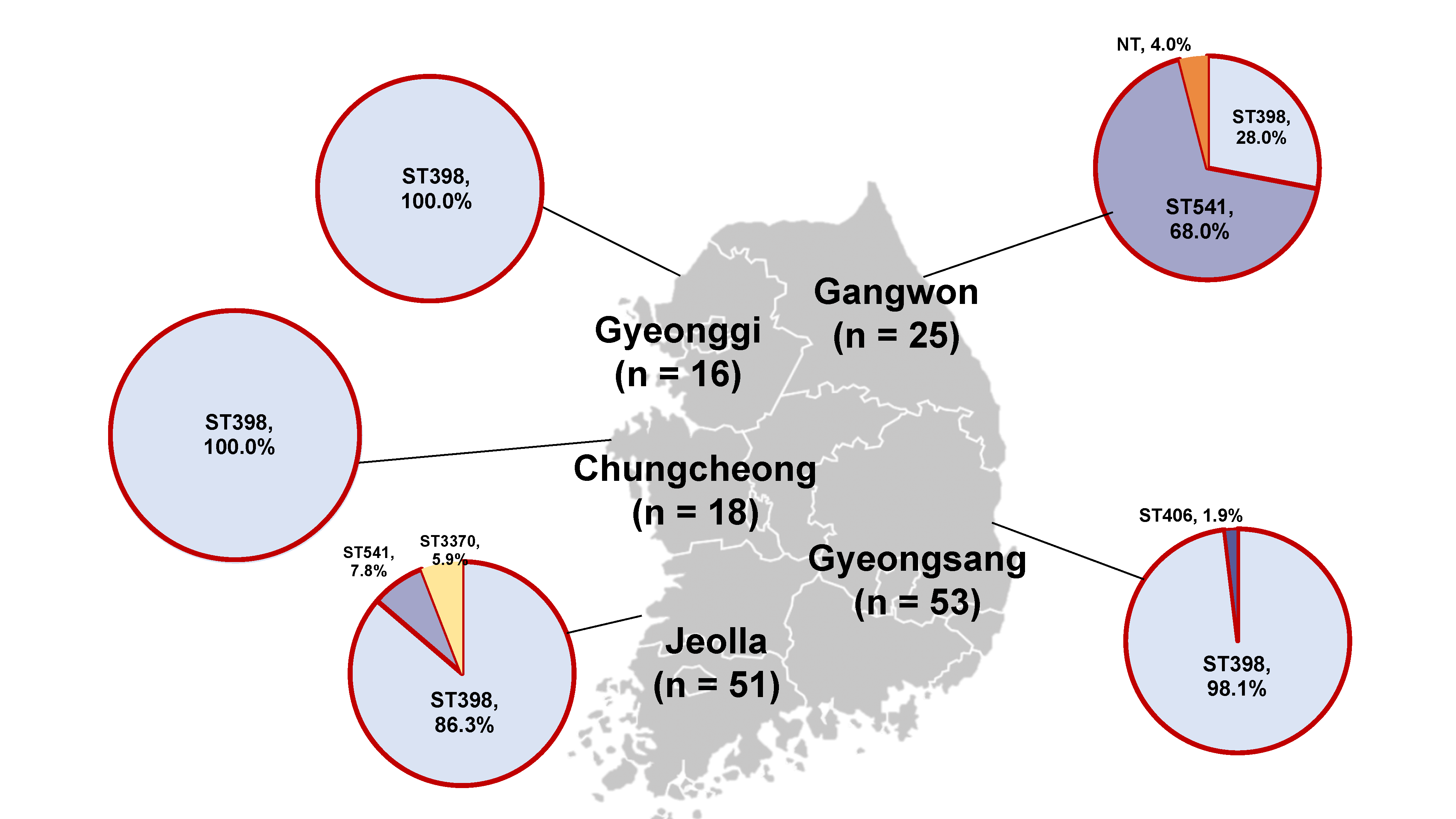

Supplement: Supplementary file 2 — Supplementary Material 2 [file 12917_2024_4360_MOESM2_ESM.tif]
