# Supplementary material for: Genetic characteristics and antimicrobial resistance of Staphylococcus aureus isolates from pig farms in Korea: emergence of cfr-positive CC398 lineage
Source: BMC Vet Res. 2024 Nov 1;20:503. doi: 10.1186/s12917-024-04360-w (PMC11529005; doi:10.1186/s12917-024-04360-w)
Supplement: Supplementary file 3 — Supplementary Material 3 [file 12917_2024_4360_MOESM3_ESM.docx]

**Table S1. Oligonucleotide primer sequences used in this study**

| Target genes | Primer name | Sequences (5’→3’) | Ref. |
| --- | --- | --- | --- |
| *mecA* | mecA-F | TGTATGGCATGAGTAACGAA | In this study |
|  | mecA-R | AAGGGAGAAGTAACAGCACT |  |
| *ccr* gene complex | ccrA1-609-628 | CAAGCCTTATCAGGTACGAA | [21] |
|  | ccrA2-1136-1155 | CATTACGTCAACAACCGCAA |  |
|  | ccrA3-1106-1127 | CTGAATCATTCAGAAAACAGAC |  |
|  | ccrA4-1121-1141 | GTCCTAAAACAGCAACAAATGA |  |
|  | ccrB1-375-356 | GAGCATTAACTTGCCTGTTG |  |
|  | ccrB2-72-51 | CCTTCTGTGCTTTGCATTTC |  |
|  | ccrB3-223-204 | GACCTTCGTTCGCATCTTTT |  |
|  | ccrB4-68-49 | GGTTACAGTATTCAAGGTCAAT |  |
|  | ccrB6-505-481 | CTGGTATGTTATTATATCCTAAAG |  |
|  | ccrC-181-202 | GCAATGAAACGTCTATTACAAG |  |
|  | ccrC-382-361 | CAAACATTGTAACGAGTACTTC |  |
| *mec* gene  complex | mecA_univ-149-126 | CTGCTATCTTTATAAACTTGTTTG |  |
|  | mecA_E-758-737 | ACATAACCTAAAAGGTGTACTG |  |
|  | mecR1_B-987-966 | TCATGTGAAGCTCGATATACT |  |
|  | mecR1_E-121-98 | ACCAAACTTTATGATTTAACTGAG |  |
|  | mecR1_A-1110-1087 | TTCATTATAAAGCACAAAACTTCC |  |
|  | IS431_C2-103-82 | GTTGAATGATGAACGTTTACAC |  |
|  | IS431_C1-254-275 | GGGACATACATTAGATATTTGG |  |
| *cfr* | cfr detection-F | GCAAACGAAGGGCAGGTAGA | In this study |
|  | cfr detection-R | TTCCACCCAGTAGTCCATTCA |  |
|  | cfr seq-F | GCGAAATGGCTCAATTTTCA | [22] |
|  | cfr seq-R | TTCCACCCAGTAGTCCATTCA |  |
| *optrA* | optrA detection-F | AACGCAAAGGAGGATATGAAAA | In this study |
|  | optrA detection-R | TCAAGGTGGTTAGTAGGTTCATCA |  |
| *fexA* | fexA detection-F | GTACTTGTAGGTGCAATTACGGCTGA | [53] |
|  | fexA detection-R | CGCATCTGAGTAGGACATAGCGTC |  |
